# Endoscopic pyloroplasty for severe gastric outlet obstruction due to alkali ingestion in a child 

**Published:** 2016

**Authors:** Seyed Mohsen Dehghani, Mitra Aldaghi, Hazhir Javaherizadeh

**Affiliations:** 1 *Shiraz Transplant Research Center, Gastroenterohepatology Research Center, Nemazee Teaching Hospital, Shiraz University of Medical Sciences, School of Medicine, Shiraz, Iran*; 2 *Department of Pediatrics, Sabzevar University of Medical Sciences, Sabzevar, Iran*; 3 *Faculty of Medicine, Ahvaz Jundishapur University of Medical Sciences, Ahvaz, Iran*

**Keywords:** Endoscopic pyloroplasty, Obstruction, Gastric injury

## Abstract

A common belief is that alkali ingestion causes severe esophageal damage and limited gastric injury due to the buffering action of acid. Gastric injury has been observed in patients who ingested alkali. Gastric outlet obstruction (GOO) secondary to caustic ingestion occurs due to fibrosis after resolution of the acute injury and inflammation, most commonly 6 to 12 weeks after initial ingestion. The traditional treatment for GOO related to ingestion of corrosive agents is surgery. Experience with endoscopic balloon dilation of corrosive-induced GOO is limited in children. This is the first report of endoscopic pyloroplasty in a child with GOO due to caustic alkalis ingestion that was treated with balloon dilation (using TTS balloon ranging from 6-15 mm) in Iran. Four dilation sessions were required for symptomatic relief of dysphagia. After one year of follow up, weight gain was normal.

## Introduction

 Caustic ingestion is seen most often in young children (1). A common belief is that alkali ingestion causes severe esophageal damage and limited gastric injury due to the buffering action of acid. However, in one report, gastric injury was observed in 94 percent of 31 patients who ingested alkali (2). Pyloric stenosis can occur with both acids and alkalis caustic ingestion and is often associated with esophageal injury and strictures. With a severe injury to the stomach, gastric outlet obstruction (GOO) may occur as early as three weeks or as late as 10 weeks. The traditional treatment for GOO related to ingestion of corrosive agents has been surgery (3). Surgical bypass may be necessary, but endoscopic balloon dilatation has also been used successfully in adults (4, 5). In children, there is a limited experience with endoscopic balloon dilation of the pylorus (pyloroplasty) (6). In this case report we presented a 1.5-year-old girl with GOO due to caustic ingestion that was resolved after balloon dilatation. 

## Case Report

A 1.5-year-old girl was admitted to the emergency department following accidental ingestion of caustic material. The caustic liquid in an unlabeled container was mistaken for water. Past medical history was normal. Immediately after ingestion she was given a cup of water by her mother and then developed a cough and vomiting. A small amount of coffee ground was noted in vomiting contents. On arrival to the pediatric emergency department, she was febrile and irritable, but hemodynamically stable without respiratory distress. Drooling was noted during inspection. Oral cavity was erythematous with some aphthus ulcers. Abdomen was soft with no tenderness or distension, and had normal bowel sounds. Other physical examination was unremarkable. On laboratory examinations the complete blood count was normal with white blood cells of 12800/mm^3^, hemoglobin of 11.7 g/dL, and platelets of 383000/mm^3^. Renal function and serum electrolytes were in normal range. Intravenous (IV) fluids and IV pantoprazole 10 mg twice per day, as well as IV antibiotics were initiated.

**Figure 1:**
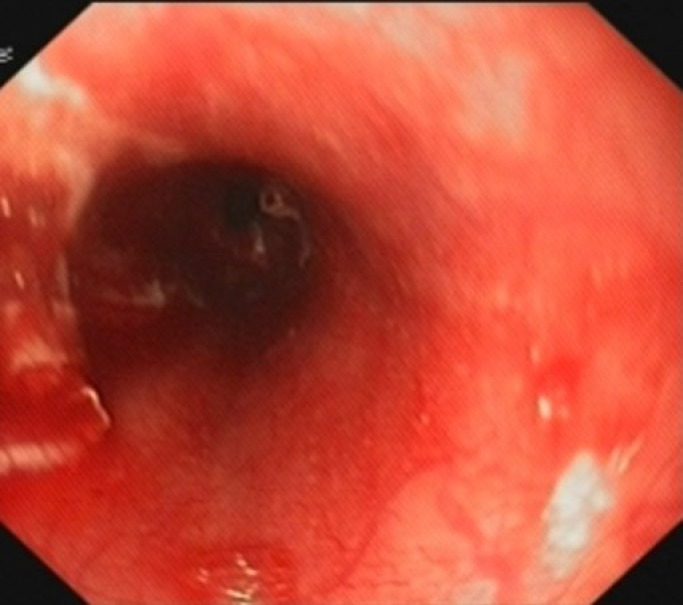
Middle third of Esophagus

Upper gastrointestinal endoscopy with general anesthesia was done to assess the severity of the caustic injury. This revealed an erythematous oral cavity with some aphthous ulcers, and grade 2B of caustic burn in esophagus (Figure 1).

**Figure 2:**
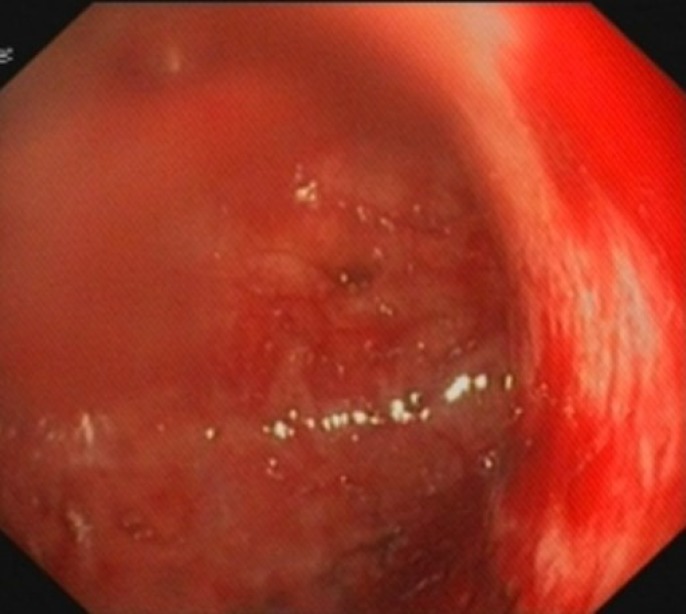
Antrum

The stomach revealed grade 2 diffuse caustic mucosal injury of the antrum and pylorus characterized by superficial erosions with exudates and edema (Figure 2). The duodenum had a normal appearance.

**Figure 3:**
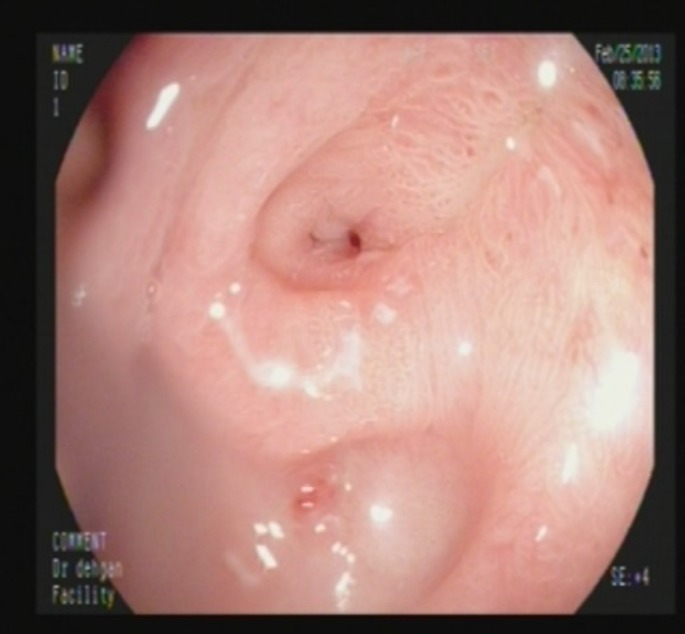
Pyloric stenosis after one month

**Figure 4:**
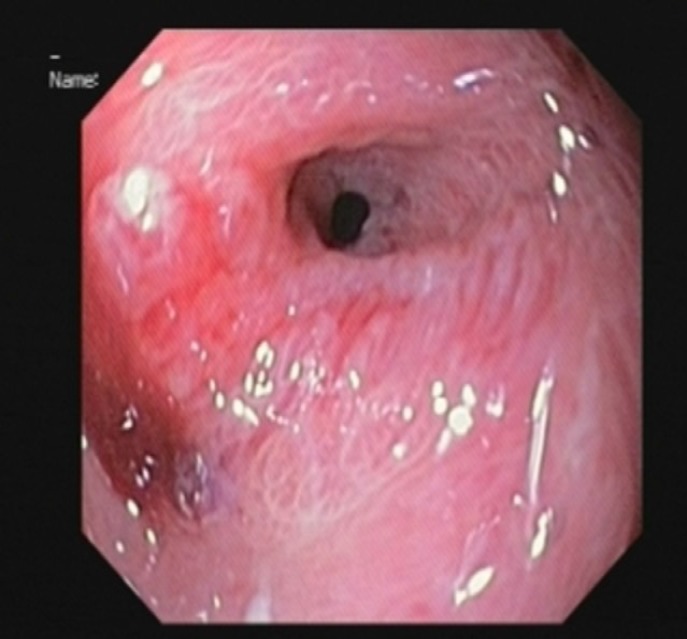
Pyloric stenosis after 2 months

**Figure 5:**
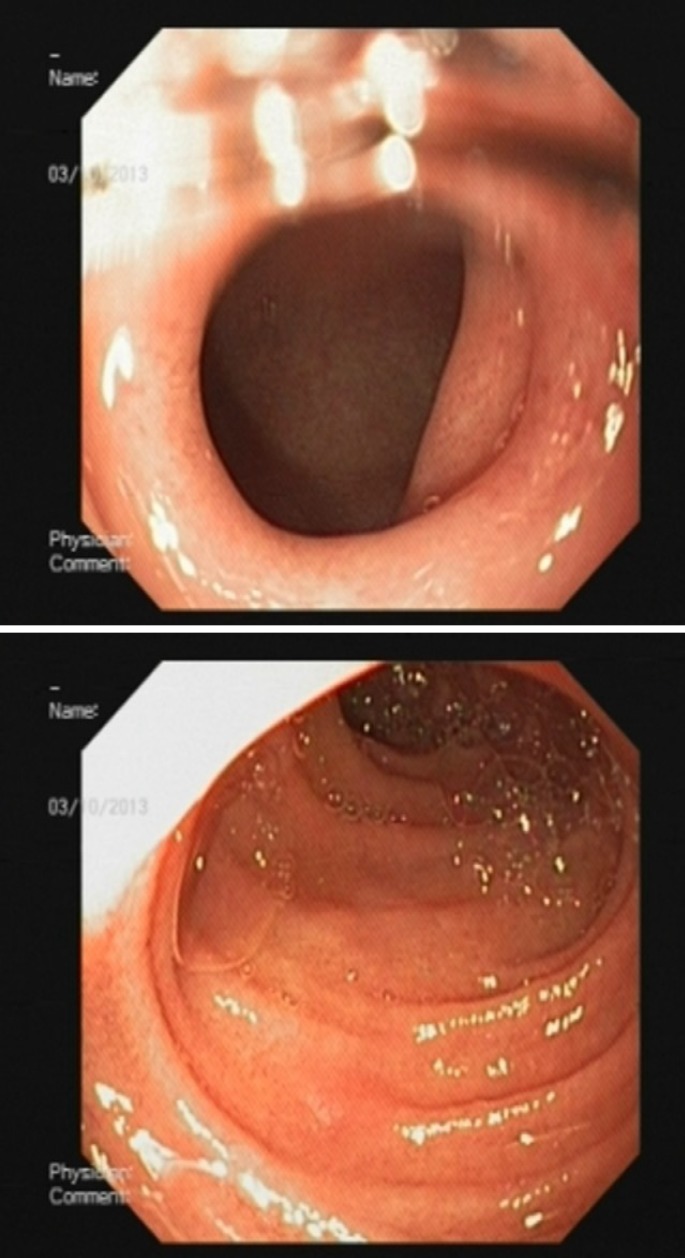
Normal pyloric canal and duodenum after one year

She kept NPO for 48 hours and then surgical diet was started without any problems. Her diet was subsequently advanced to liquid and soft, and diet was well tolerated. After 4 weeks of follow up, she developed repeated vomiting after feeding. Upper gastrointestinal endoscopy was performed again and revealed a normal oral cavity and esophagus, but deformed antrum with severe stricture in pylorus where a 6mm scope could not pass through it (Figure 3). After a parental agreement for this method, the patient was prepared for balloon dilatation. Guide wire was inserted through endoscopic lumen. The balloon was passed over the guide wire through the pylorus. Balloon dilation was done by TTS (Through the Scope) balloon dilator. After 4 courses of balloon dilatation with a 6-15 mm balloon with 3-4 weeks interval, the pyloric canal became normal and the scope passed easily into the duodenum (Figure 4). The patient was well and symptom free with normal feeding and weight gain after more than one year follow up. The normal pyloric canal was seen after management (Figure 5). 

## Discussion

Caustic ingestion is seen most often in children between one and three years of age with elevated rates in boys (7, 8). Esophageal damage from ingestion of caustic liquids, either acidic or alkaline, is more common than gastroduodenal damage. 

Gastrojejunostomy was used as an effective method for treatment in some centers in isolated corrosive pyloric stenosis (9). There are limited reports of endoscopic balloon dilation as a treatment. 

Surgical bypass may be necessary, but endoscopic balloon dilatation has also been used successfully in patients with pyloric stenosis due to caustic ingestion (6). Temiz et al. reported 7 cases with corrosive stricture, and successful balloon dilation was performed in 5 of them. After a short period of time, three patients showed evidence of stricture (6). Experience with endoscopic balloon dilation of corrosive-induced GOO is limited. The traditional treatment for GOO related to ingestion of corrosive agents is surgery.

In conclusion, endoscopic balloon dilation is a less invasive and interesting method of treatment in children with pyloric stricture due to caustic ingestion. However, more experiences will be required in endoscopic balloon dilation among children with pyloric stricture to avoid more invasive procedures.
